# Comparison of livestock-associated and community-associated *Staphylococcus aureus* pathogenicity in a mouse model of skin and soft tissue infection

**DOI:** 10.1038/s41598-019-42919-y

**Published:** 2019-05-01

**Authors:** Pranay R. Randad, Carly A. Dillen, Roger V. Ortines, David Mohr, Maliha Aziz, Lance B. Price, Hülya Kaya, Jesper Larsen, Karen C. Carroll, Tara C. Smith, Lloyd S. Miller, Christopher D. Heaney

**Affiliations:** 1 Department of Environmental Health and Engineering, Johns Hopkins Bloomberg School of Public Health, Baltimore, Maryland USA; 2 Department of Dermatology, Johns Hopkins School of Medicine, Baltimore, Maryland USA; 3 Genetic Resources Core Facility, McKusick-Nathans Institute of Genetic Medicine, Johns Hopkins University School of Medicine, Baltimore, Maryland USA; 4 Department of Environmental and Occupational Health, George Washington University, Washington, D.C. USA; 5 Antibiotic Resistance Action Center, George Washington University, Washington, D.C. USA; 6 Department of Bacteria, Parasites and Fungi, Statens Serum Institut, Copenhagen, Denmark; 7 Division of Medical Microbiology, Johns Hopkins University School of Medicine, Baltimore, Maryland USA; 8 Department of Epidemiology and Biostatistics, Kent State University, Kent, Ohio, USA; 9 Department of Epidemiology, Johns Hopkins Bloomberg School of Public Health, Baltimore, Maryland USA; 10 Department of International Health, Johns Hopkins Bloomberg School of Public Health, Baltimore, Maryland USA

**Keywords:** Infection, Bacterial infection

## Abstract

Industrial hog operation (IHO) workers are at increased risk of carrying *Staphylococcus aureus* in their nares, particularly strains that are livestock-associated (LA) and multidrug-resistant. The pathogenicity of LA-*S. aureus* strains remains unclear, with some prior studies suggesting reduced transmission and virulence in humans compared to community-associated methicillin-resistant (CA-MRSA) *S. aureus*. The objective of this study was to determine the degree to which LA-*S. aureus* strains contracted by IHO workers cause disease relative to a representative CA-MRSA strain in a mouse model of skin and soft tissue infection (SSTI). Mice infected with CC398 LA-*S. aureus* strains (IHW398-1 and IHW398-2) developed larger lesion sizes with higher bacterial burden than mice infected with CA-MRSA (SF8300) (*p* < 0.05). The greatest lesion size and bacterial burden was seen with a CC398 strain that produced a recurrent SSTI in an IHO worker. The LA-*S. aureus* infected mice had decreased IL-1β protein levels compared with CA-MRSA-infected mice (*p* < 0.05), suggesting a suboptimal host response to LA-*S. aureus* SSTIs. WGSA revealed heterogeneity in virulence factor and antimicrobial resistance genes carried by LA-*S. aureus* and CA-MRSA strains. The observed pathogenicity suggest that more attention should be placed on preventing the spread of LA-*S. aureus* into human populations.

## Introduction

In the past decade it has become evident that animal-adapted multidrug resistant *Staphylococcus aureus* (MDRSA) has emerged among food animals raised in concentrated animal feeding operations (CAFOs) and individuals who have frequent contact with food animals raised in CAFOs globally^1,2^, including the United States^3–5^. A study conducted in Pennsylvania concluded that residential proximity to swine CAFO manure land application crop fields was associated with increased odds of methicillin-resistant *S. aureus* (MRSA) infection and skin and soft tissue infection (SSTI)^6^, suggesting an environmental exposure pathway wherein community members could become infected with antimicrobial-resistant *S. aureus* originating at swine CAFOs. This is consistent with evidence suggesting that industrial hog operation (IHO) workers are at an increased risk of carrying livestock-associated (LA) *S. aureus*, including LA-MDRSA, intranasally^4,7–9^. LA-MDRSA originating at IHOs may also be carried and transmitted from IHO workers to family contacts, noted by an increased prevalence of MRSA and MDRSA nasal carriage among children living with IHO workers in North Carolina compared to children living with community resident adults with no livestock exposure^10^. Less is known about the extent to which nasal carriage of such LA-*S. aureus* is associated with infection, particularly skin and soft tissue infections (SSTIs).

While large-scale surveillance studies from Europe suggest that LA-MRSA strains are capable of causing the full suite of human infections^11^, some reports suggest that these strains display a decreased capacity for human-to-human transmission and may be less pathogenic than typical community associated (CA)- and hospital associated (HA)-*S. aureus* strains^12–16^. USA300 is a hypervirulent clone of *S. aureus* that emerged in the USA in the late 1990’s, and has become the dominant CA-MRSA strain circulating in North America^17^. Consistent with its ability to cause severe and widespread disease, USA300 clone, SF8300, displayed considerably increased virulence in a mouse model of skin and soft tissue infection (SSTI) compared to other MRSA lineages^18^.

At a genetic level, LA-*S. aureus* tend to lack genetic factors associated with human infection that are typically found in CA-MRSA lineages, including the human immune evasion cluster (IEC) genes (*scn*) and Panton-Valentine leukocidin (PVL)-encoding genes (*lukS-PV* and *lukF-PV*)^13,19,20^. Nevertheless, LA-*S. aureus* strains have been reported to produce skin and bloodstream infections in humans in Europe^11,21–24^, the USA^7,9,25–27^, and Canada^18^. A recent study concluded that a LA-MDRSA isolate collected from poultry displayed greater lethality in a murine sepsis model compared to a clinical methicillin-susceptible *S. aureus* (MSSA) isolate and provided information on differential gene expression^28^. To our knowledge, no studies have assessed the relative pathogenesis of LA-*S. aureus* acquired from swine on an IHO compared to a well-characterized and highly pathogenic CA-MRSA strain isolated from a human SSTI outbreak in the community. Considering that LA-*S. aureus* has surfaced in community members, including children, who experience environmental exposure to CAFOs, it is critical to improve our understanding of the pathogenic potential of LA-*S. aureus* strains emerging from CAFO environments and resulting in human SSTI. This study aims to understand the degree to which LA-*S. aureus* strains contracted by IHO workers cause disease relative to a representative hypervirulent CA-MRSA strain–*i.e*., USA300 clone, SF8300^29,30^, in a mouse model of SSTI.

## Methods

### Selection of *S. aureus* isolates

One CC9 and one CC8 LA-MDRSA strain were selected that were collected from the anterior nares of two IHO workers who reported a SSTI within the past 3 months between October 2013 and February 2014 in a prospective cohort study of IHO workers in North Carolina (NCHW9 and NCHW8)^7^. Two CC398 LA-MDRSA isolates were selected that were collected directly from two IHO workers’ active skin infections between May 2011 and February 2013 in a prospective cohort study of IHO workers in Iowa^9^, and were provided by Dr. Tara C. Smith from Kent State University (IHW398-1 and IHW398-2). IHW398-1 was responsible for a physician-diagnosed recurrent SSTI in a male hog worker^27^. The representative CA-MRSA strain SF8300 was isolated from a SSTI in a patient treated at the San Francisco General Hospital and was provided by Dr. Henry Chambers of University of California San Francisco. SF8300 is pulsed-field type USA300, which responsible for the vast majority of CA-MRSA SSTI in the USA, Canada, and Europe^29^, and displays a high degree of pathogenicity in a mouse model of SSTI^30^. Hemolytic activity was treated as a binary variable and was confirmed for each isolate by observation of a zone of hemolysis after 24-hours of growth on sheep blood agar plates.

### Indicators of livestock association

There is currently no established molecular marker for LA-*S. aureus*. LA-MDRSA has been consistently classified as: (i) MDRSA carriage or infection in humans arising from exposure to livestock, (ii) belonging to the clonal complex 398 (CC398) or CC9, and (iii) lacking the staphylococcal complement inhibitor gene *scn* (*scn-)*. Here we used CC9 or CC398 within the IIa livestock clade of the CC398 phylogeny, and the absence of the *scn* gene (*scn-*) as indicators of LA-*S. aureus*. Putative clonal complex (CC) was previously assigned to each isolate based on *spa type*, using the Ridom StaphType software and the Ridom SpaServer (http://spa.ridom.de/index.shtml).

### Antimicrobial susceptibility testing

Each selected *S. aureus* strain was subjected to a panel of antibiotics for antibiotic susceptibility testing (AST) using the Phoenix Automated Microbiology System (BD Diagnostic Systems, Sparks, MD) by the the Clinical Microbiology Laboratory at the Johns Hopkins Hospital, according to guidelines for clinical isolates. MRSA was defined as resistant to cefoxitin or oxacillin and positive for the *mecA* or *mecC* gene. MDRSA was defined as resistant to greater than or equal to three classes of antibiotics. MIC cut off’s used to establish resistant, intermediate, or susceptible phenotypes, and antibiotic abbreviations, are provided in Chapter 3: Supplementary Information (Table S1).

### *S. aureus* growth curves

*S. aureus* strains were streaked onto tryptic soy agar plates and grown overnight. Single colonies were selected and grown in tryptic soy broth (TSB) at 37 °C in a shaking incubator overnight, shaking at 240 rpm and then sub-cultured at a 1:50 dilution in TSB. At 0, 0.5, 1, 1.5, 2, 2.5, 3, 3.5, 4, and 4.5 hours, 100 µL of the sub-cultures were pipetted onto a 96-well plate and absorbance (600 nm) was read with a Synergy H1 Hybrid Microplate Reader (BioTek Instruments, Inc., Winooski, VT).

### Mouse model of *S. aureus* skin and soft tissue infection

Animal care and all experiments were approved and performed in accordance with the guidelines and regulations approved by the Johns Hopkins University Animal Care and Use Committee, which conform to the Guide for the Care and Use of Laboratory Animals published by the US National Institutes of Health (8^th^ edition, 2011). As a first step toward identifying the relative pathogenicity of LA-*S. aureus* compared to that of CA-MRSA, we evaluated the skin lesions that developed in response to intradermal (i.d.) infection of four LA-MDRSA isolates collected from IHO workers and a representative CA-MRSA strain (SF8300) in a mouse model of SSTI (Fig. 2A). The SF8300 strain was chosen as a representative CA-MRSA strain for this study because it is the same pulsed-field USA300 type responsible for the epidemic of CA-MRSA SSTI in humans in the USA^29^ and has been previously used to compare pathogenicity of *S. aureus* cutaneous infections in mice^30^. The upper backs of C57BL/6 mice were shaved and inoculated intradermally with 3 × 10^7^ colony-forming units (CFU) in 100 µl PBS of midlogarithmic growth phase SF8300 (CC8) (n = 20 mice) or the following LA-*S. aureus* strains (n = 10 mice/strain) NCHW8 (CC8), NCHW9 (CC9), IHW398-1 (CC398), or IHW398-2 (CC398). Digital photographs of mice taken on days 0, 1, 3, 7, 10, and 14 and analyzed for measurements of total lesion size (cm^2^) using ImageJ software. Data reported as mean total lesion size (cm^2^) ± the standard error of the mean (SEM).

### Measuring bacterial burden

Mice (n = 5/group) were euthanized on day 3 post-infection and 10-mm skin punch biopsies of lesions were homogenized (Pro200 Series homogenizer; Pro Scientific, Oxford, CT) in PBS on ice. Samples were serially-diluted and cultured on TSA plates overnight and CFU were enumerated.

### Cytokine, chemokine, and growth factor evaluation in infected skin

Infected skin biopsies from day 3 were analyzed for protein levels of cytokines, chemokines and growth factors to provide insights into the host response to CA-MRSA or LA- *S. aureus* i.d. inoculation. Mice (n = 5/group) were euthanized on day 3 post-infection and 10-mm skin punch biopsies of lesions were weighed and snap-frozen in liquid nitrogen. On ice, each specimen was homogenized with a hand-held homogenizer (Pro200 Series homogenizer; Pro Scientific) in Protein Lysis Buffer (Promega) containing protease inhibitor cocktail (Roche). All samples were stored at 80 °C. Samples were then centrifuged at 4 °C and supernatants were assayed for protein levels of cytokines, chemokines and growth factors using a 9-plex and 11-plex mouse protein array, according to the manufacturer’s recommendations (Bio-Plex Pro™, Biorad; Hercules, CA). For the 9-plex and 11-plex arrays, samples were normalized to 0.75 mg/mL and 2 mg/mL total protein, respectively. Samples were also assayed for myeloperoxidase (MPO) levels using a commercially available ELISA kit (R&D Systems, Minneapolis, MN).

### Genome sequencing

We sequenced and assembled the genomes of the LA-MDRSA isolates, and used the publicly available genomic sequence of SF8300^29^ for WGSA. DNA was prepared for multiplexed, paired-end sequencing on an Illumina MiSeq (Illumina, Inc., San Diego, CA). For each isolate, 500 ng of DNA was sheared to an average fragment size of 300 bp in 50 µL using a Covaris E220 Focused-Ultrasonicator (Covaris, Woburn, MA). End repair, A-tailing, and adaptor ligation of the total volume of sheared DNA was performed using the Kapa Hyper Prep Kit (Kapa Biosystems, Inc., Wilmington, MA) with recommended adaptor concentrations for libraries constructed from 500 ng input DNA. Uniquely barcoded adaptors used in adaptor ligation were obtained from BioO Scientific® (BioO Scientific®, Austin, TX, NEB, NEXTflex-96™ DNA Barcodes for DNA). Following ligation of adaptors, KAPA Pure Beads (Kapa Biosystems, Inc., Wilmington, MA) were used to perform a 0.8X bead-based cleanup for each DNA library. Individual libraries were quantified in triplicate at two concentrations (1:100 and 1:1000) via quantitative PCR using the Kapa Library Quantification kit (Kapa Biosystems, Inc. Wilmington, MA,). Based on individual library concentrations, equimolar pools of *S. aureus* libraries were prepared at a concentration of at least 1 nM. The pooled libraries were qc’d on an Agilent bioanalyzer and sequenced on an Illumina MiSeq at 2 × 300 bp.

### *De novo* assembly and molecular characterization of *S. aureus* genomes

To gain information on the genetic basis for the pathogenic potential of LA-MDRSA as compared to SF8300, we assessed the presence of core and mobile genetic element (MGE) encoded virulence factors (VF’s) and acquired antimicrobial resistance (AMR) genes important for pathogenesis in humans. Illumina short-read sequences were trimmed using trimmomatic^31^, and assembled into contigs using the SPADES assembler (v.3.5)^32^. Assembly quality was assessed using QUAST (v.2.3)^33^, and reference MLST housekeeping genes were identified using BLAST (Version 2.2.25+)^34^ at 100% query coverage and nucleotide identity (ID). An in-house script was used to match genes and MLST profile data^35^ to determine the final MLST type. These analyses were performed on the GWU Colonial High Performance Computing Cluster. Virulence factor (VF) and Antimicrobial resistance (AMR) genes were determined by uploading each assembled *S. aureus* genome onto the *S. aureus* VirulenceFinder 1.5^36^ and ResFinder 3.0^37^, available on the Center for Genomic Epidemiology (CGE) server. ABRicate (https://github.com/tseemann/abricate), a modified BLASTn based tool for the screening of genes in assemblies, was used with a custom database containing microbial surface components recognizing adhesive matrix molecules (MSCRAMMs), and several additional hemolysins and leucocidins, to detect the following genes on the assembled *S. aureus* genomes: *clfA* (GenBank accession no. Z18852) and *clfB* (GenBank accession no. AJ224764) encoding clumping factor A and B (ClfA and ClfB), *sdrC* (GenBank accession no. AJ005645), *sdrD* (GenBank accession no. AJ005646), and *sdrE* (GenBank accession no. AJ005647) encoding serine-aspartate repeat protein C, D, and E (SdrC, SdrD, and SdrE), *bbp* (GenBank accession no. BX571856) encoding bone sialoprotein-binding protein (Bbp), *fnbpA* (GenBank accession no. J04151) and *fnbpB* (GenBank accession no. X62992) encoding Fibrinogen-binding protein A and B (FnBPA and FnBPB), and *cna* (GenBank accession no. M81736) encoding collagen adhesin (Cna), *hla* (GenBank accession no. CP000255, BX571856, BX571857, BA000017, BA000033, BA000018, CP000046) encoding for alpha hemolysin, *hlb* (GenBank accession no. BX571856, BX571857, BA000017, BA000033, BA000018, CP000046) encoding for beta hemolysin, *lukA* and *lukB* (GenBank accession no. AP009351) encoding for the LukAB leucocidin, and *nor* (GenBank accession no. BX571856) encoding for nitric oxide reductase. For all genome hits, the threshold of ID was set to 85% and percentage of minimum gene length was set to 60%. All genome hits were manually inspected for confirmation.

### Statistical analysis

Data were compared using Student’s *t* test (two-tailed), comparing each LA-*S. aureus* strain to the representative SF8300 CA-MRSA referent strain. *p*-values < 0.05 were considered statistically significantly.

## Results

### LA-*S. aureus* and antibiotic susceptibility testing

**Figure 1:**
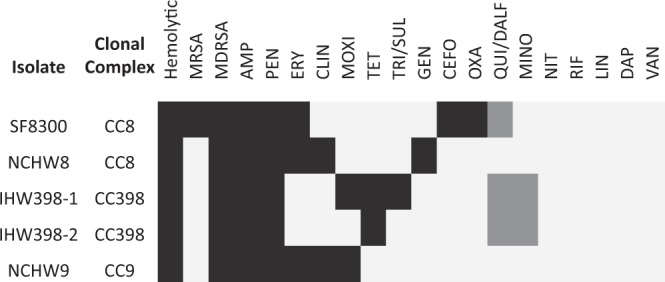
Hemolytic activity and antibiotic susceptibilities of LA-*S. aureus* and CA-MRSA. Hemolysis and antibiotic susceptibility testing results are shown for each *S. aureus* isolate Hemolytic activity was measured as a binary variable, and defined as the formation of a zone of hemolysis after 24 hours of growth on blood sheep agar. MRSA was defined as cefoxitin or oxacillin resistant or positive for the *mecA* or *mecC* gene. MDRSA was defined as resistant or intermediate to at least three classes of antibiotics. For the hemolytic, MRSA, and MDRSA categories, black = positive and white = negative. For antibiotic susceptibility categories, black = resistant, grey = intermediate, and white = susceptible. *Note*. AMP = ampicillin, PEN = penicillin, ERY = erythromycin, CLIN = clindamycin, MOXI = moxifloxacin, TET = tetracycline, TRI/SUL = trimethoprim/sulfamethoxazole, GEN = gentamicin, CEFO = cefoxitin, OXA = oxacillin, MINO = minocycline, NIT = nitrofurantoin, RIF = rifampicin, LIN = linezolid, DAP = daptomcyin and VAN = vancomycin.

Of the 17 antibiotics tested, complete or intermediate resistance was observed to all antibiotics except for vancomycin, linezolid, daptomycin, rifampicin, and nitrofurantoin (Fig. 1). All *S. aureus* clones were resistant to ampicillin and penicillin, and displayed a multidrug resistant phenotype, while only the CA-MRSA clone SF8300 displayed a methicillin-resistant phenotype (Fig. 1). The SF8300 isolate also displayed complete resistance to erythromycin and cefotaxime, and intermediate resistance to quinupristin/dalfopristin (Fig. 1). The NCHW8 isolate, collected from an IHO worker’s nares, additionally displayed resistance to erythromycin, clindamycin, and gentamycin (Fig. 1). Both CC398 isolates displayed resistance to tetracycline and intermediate resistance to quinupristin/dalfopristin and minocycline, while the IHW398-1 isolate, responsible for a recurrent SSTI in an IHO worker, also displayed resistance to moxifloxacin and sulfamethoxazole/trimethoprim (Fig. 1). The SSTI-associated CC9 isolate displayed resistance to erythromycin, clindamycin, and moxifloxacin (Fig. 1).

### LA-*S. aureus*-infected mice and skin lesion size

**Figure 2:**
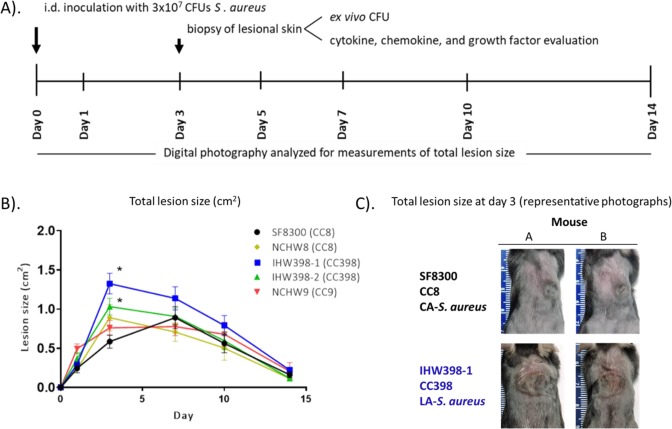
LA-*S*. aureus-infected mice develop larger lesion sizes compared to CA-MRSA. (**A**) Schematic of mouse model of SSTI. (**B**) Mean total lesion size (cm^2^) ± SEM. (**C**). Representative photographs of the lesions at day 3 for two mice. 1*, *p* < 0.05 = each LA-*S. aureus* strain (n = 10/group) versus CA-MRSA SF8300 (n = 20), as calculated by a two-tailed Student’s *t-*test. *Note*. LA = livestock-associated. CA = community-associated. MRSA = methicillin-resistant *S. aureus*. i.d. = intradermal. CFU = colony forming unit.

Growth curves showed a mid-logarithmic phase at 3 hours for all isolates, and there were no statistically significant differences in growth curves between isolates (Fig. 2M.1). Mice were inoculated i.d. with 3 × 10^7^ CFU’s of mid-logarithmic growth phase *S. aureus* and skin lesion sizes were evaluated over time (Fig. 2A). All of the *S. aureus* strains resulted in visible skin lesions that healed by day 14. SF8300 and NCHW9 infected mice developed maximum lesion size on day 7 of 0.9 ± 0.1 cm^2^ and 0.8 ± 0.1 cm^2^, respectively. IHW398-1, IHW398-2, and NCHW8 infected mice developed maximum lesion sizes on day 3 of 1.3 ± 0.1 cm^2^, 1.0 ± 0.1 cm^2^, and 0.9 ± 0.2 cm^2^, respectively. Both CC398 LA-MDRSA strains collected directly from IHO workers with active SSTI developed significantly larger lesion sizes on day 3 compared with SF8300 (*p* < 0.05) (Fig. 2B). The skin lesions sizes of all other LA-MDRSA isolates (CC9 or CC8) were not statistically different from those of SF8300 at any time point. Of note, mice infected with the IHW398-1 clone which was responsible for a recurrent SSTI in an IHO worker, developed the largest lesion sizes, peaking on day 3 and were 2-fold greater than those of SF8300-infected mice (Fig. 2C).

### LA-*S. aureus*-infected mice and bacterial burden

**Figure 3:**
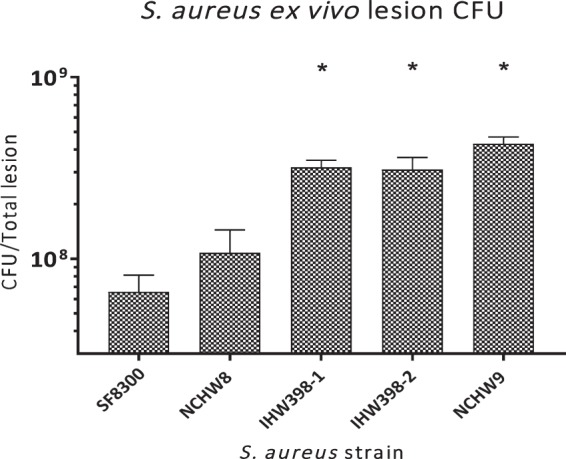
*Ex vivo* colony forming units (CFU) in LA-*S. aureus* and CA-MRSA infected skin. On day 3, homogenates of infected skin were assayed for *ex vivo* CFU levels. *Ex vivo* CFU ± SEM. *p < 0.05, between LA-*S. aureus* strains versus SF8300 (n = 5/group), as calculated by a two-tailed Student’s *t-*test.

Given that the CC398 lesion sizes peaked on day 3, we evaluated the bacterial burden by determining *ex vivo* CFU isolated from skin punch biopsy specimens of the entire skin lesions obtained on day 3. *Ex vivo* CFU from CC398 (IHW398-1 and IHW398-2) and CC9 LA-MDRSA*-*infected mice were significantly greater compared with SF8300-infected mice (Fig. 3), suggesting increased bacterial proliferation and/or persistence of these particular LA-MDRSA strains *in vivo*.

### LA-*S. aureus*-infected mice and local cytokine, chemokine, and growth factor levels

**Figure 4:**
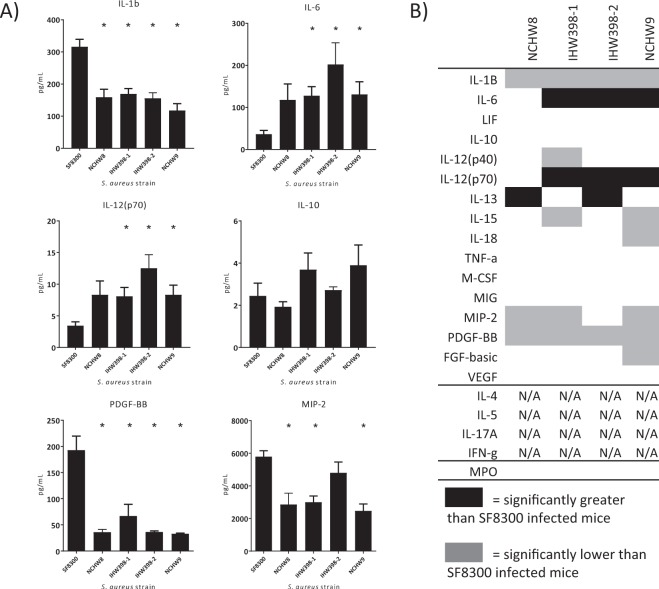
Cytokine, chemokine and growth factor protein levels in LA-*S. aureus* and CA-MRSA infected skin. On day 3, homogenates of infected skin were assayed for *ex vivo* protein levels of host response proteins. (**A**) Mean protein levels (pg/mL homogenate) ± SEM. *p < 0.05, between LA-*S. aureus* strains versus SF8300, as calculated by a two-tailed Student’s *t-*test. (**B**) Summary of LA*-S. aureus* protein levels compared to CA-MRSA (SF8300) protein levels for all 21 proteins. *p* < 0.05 = statistically significant difference via two-tailed students *t-*test compared to reference SF8300 strain. Typically, 5 mice per *S. aureus* strain were used.

At day 3, SF8300-infected mice had significantly greater protein levels of IL-1β compared with all LA-MDRSA*-*infected mice (*p* < 0.05) (Fig. 4A). IHW398-1-, IHW398-2-, and NCHW9-infected mice had significantly greater protein levels of IL-6 compared with the CC8 strains SF8300 and NCHW8 (*p* < 0.05) (Fig. 4A). IHW398-1-, IHW398-2-, and NCHW9-infected mice also had significantly increased protein levels IL-12(p70) compared with SF8300-infected mice (*p* < 0.05) (Fig. 4A). Proteins that showed no difference between LA- and CA- *S. aureus* infected mice were IL-10 (Fig. 4A), TNF, IL-12(p40), LIF, MIG, M-CSF, VEGF, and MPO and several cytokines were below the limit of detection (IFN-γ, IL-4, IL-5, and IL-17A) (Fig. 4B). With respect to chemokines, the neutrophil-attracting chemokine MIP-2 were significantly greater in day 3 lesions of SF8300 infected mice compared to lesions of NCHW8, IHW398-1, and NCHW9 infected mice (*p* < 0.05) (Fig. 4A). Taken together, LA-*S. aureus* strains had lower IL-1β and MIP-2 but higher levels of IL-6 and IL-12(p70) compared with SF8300.

### Whole-genome sequencing analysis (WGSA) of CA- and LA-*S. aureus*

**Figure 5:**
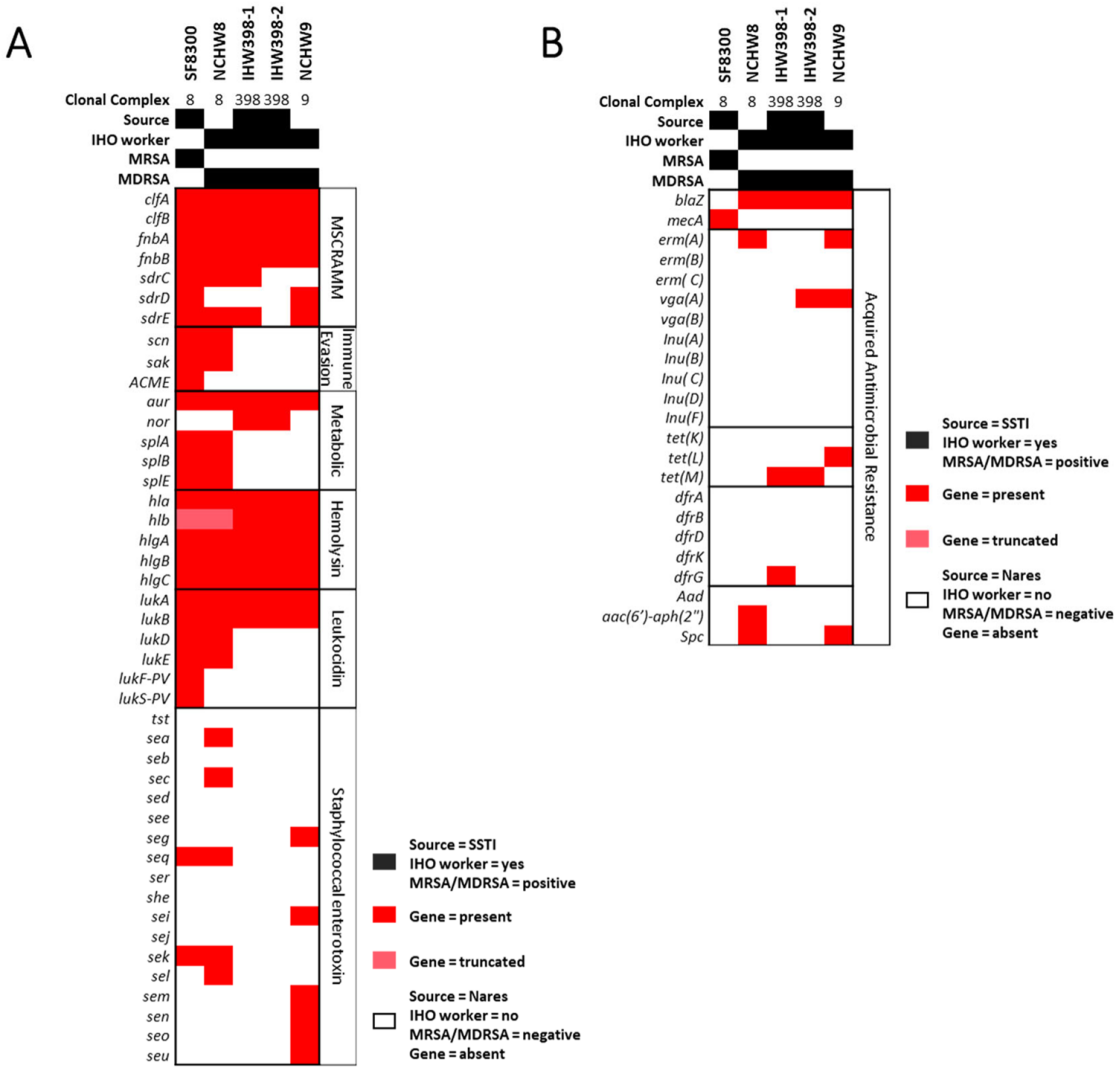
Virulence factor and AMR genes in LA-*S. aureus* and CA-MRSA. The presence of a genome hit (indicated by red shading) was defined >85% gene identification similarity and >60% gene length similarity and confirmed by manual inspection. (**A**) Microbial surface components recognizing adhesive matrix molecules (MSCRAMM), human immune evasion, metabolic enzyme, hemolysin, leucocidin/toxin, and *Staphylococcal* enterotoxin gene genome hits generated via VirulenceFinder 1.5 or ABRicate tool. (**B**) Acquired antimicrobial resistance gene genome hits generated via ResFinder 3.0. *Note*. LA = livestock-associated. CA = community-associated. MRSA = methicillin-resistant *S. aureus*. MDRSA = multidrug-resistant *S. aureus*.

Figure 5 displays genomes hits for VFs and acquired AMR genes present in at least one of the 5*S. aureus* genomes. WGSA of VF genes led to the following key findings (Fig. 5A). First, MSCRAMM genes *clfA, clfB, fnbA, and fnbB*, known to play a role host cell adhesion^38^, are present among CA- and LA- *S. aureus* strains. Second, human IEC genes *scn* and *sak* were present among CC8 isolates and were absent from LA-MDRSA CC398 and CC9 isolates. Third, the *splA, splB, and splC* genes encoding serine proteases, and reported to increase virulence *in-vivo*, present only among the CC8 strains^39^. The *nor* gene encoding for a nitric oxide reductase enzyme has also been reported to increase *S. aureus* virulence but was only present among the LA-*S. aureus* CC398 strains^40^. Fourth, hemolysin genes *hla*, known to play a critical role in dermo-necrosis^41–44^, *and the hlgA, hlgB, and hlgC* genes were conserved across CA-MRSA and LA-*S. aureus* isolates. *hlb* appeared to be truncated in the CA-MRSA strain and intact in the LA-*S. aureus* strains. Fifth, the leukotoxin encoding genes *lukD* and *lukE*, known to induce inflammation and dermonecrosis^45,46^, were present among CC8 *S. aureus* isolates, but only the SF8300 CC8 isolate carried the Panton-Valentine Leukocidin encoding genes *lukF-PV* and *lukS-PV*. *lukA* and *lukB* genes encoding for the bicomponent LukAB leucocidin were present among CA- and LA- *S. aureus* genomes. Lastly, both CC398 LA-MDRSA isolates did not contain any known enterotoxin genes typically encoded on prophages and pathogenicity islands. The genes encoding enterotoxin K (*sek)* and enterotoxin Q (*seq)* were conserved across CC8 isolates, while the SSTI-associated NCHW8 CC8 isolate additionally carried the enterotoxin A (*sea)* and enterotoxin C (*sec)* encoding genes. Unique to the CC9 LA-MDRSA isolate was the egc cluster, encoding for enterotoxin G, I, M, N, and O (*seg, sei, sem sen, seo*).

WGSA of AMR genes led to the following key findings (Fig. 5B). First, the *mecA* gene, encoding for methicillin resistance, was absent from LA-MDRSA, but these isolates carried the beta-lactamase encoding gene *blaZ*. Second, both CC398 and the CC9 LA-MDRSA genomes encoded for tetracycline resistance. The CC398 isolates carried a *tet(M)* gene, while the CC9 isolate carried a *tet(L)* gene. Third, macrolide resistance encoding genes were found only in LA-MDRSA isolates. The NCHW8 and NCHW9 isolate carried the erythromycin resistance encoding gene *erm(A)*, and the IHW398-2 and NCHW9 isolates carried the ATP-binding protein encoding gene *vga(A)* conferring resistance to streptogramin A antibiotics. Fourth, unique to the IHW398-1 isolate, responsible for a recurrent SSTI in an IHO worker, was the *dfrG* gene encoding resistance to trimethoprim antibiotics. Fifth, the *spc* gene, conferring resistance to spectinomycin, was found only in the LA-MDRSA isolates collected from IHO workers in North Carolina (NCHW8 and NCHW9). Finally, unique to the NCHW8 isolate was the *aac(6*′*)-aph(2*″) gene conferring resistance to gentamycin.

## Discussion

The results of this study demonstrate an increased degree of pathogenicity associated with LA-*S. aureus*, marked by increased lesion size and bacterial burden, compared to CA-MRSA in a mouse model of SSTI. The IHW398-1 isolate in particular, which belongs to the LA-CC398-IIa lineage and produced a recurrent SSTI in an IHO worker^27^, displayed the greatest degree of pathogenicity and a MDRSA phenotype. It is notable that the lesion size of the NCHW9 LA-CC9 isolate was equivalent to the CA-MRSA strain (SF8300). Additionally, the *S. aureus* CFU counts recovered from the NCHW9 LA-CC9 infected lesions were significantly greater than the CA-MRSA strain (SF8300) and comparable to those of the highly pathogenic IHW398-1 LA-CC398-IIa strain, suggesting that the CC9 LA-MDRSA strain displays increased bacterial proliferation and/or persistence *in vivo*. This is important because the CC9 lineage of *S. aureus* is emerging as a predominant clone among IHO workers and community residents in North Carolina^7,10^. As our efforts to understand and combat the environmental and community origins of antimicrobial-resistant *S. aureus* infections become a priority, it is critical that we continue to monitor the spread of LA-*S. aureus* into human populations and further elucidate mechanisms of virulence and pathogenicity of LA-*S. aureus* that have contributed to human morbidity.

We observed that the host cytokine and chemokine response to LA-*S. aureus* strains differed from that of CA-MRSA. Neutrophil derived IL-1β plays a critical role in amplifying and maintaining a neutrophilic response for abscess formation and bacterial clearance^47^, and previous studies have reported that *S. aureus*-infected IL-1β-deficient mice develop larger skin lesions with higher bacterial counts than wildtype mice^48^. An optimal response to *S. aureus* SSTI is associated with elevated local IL-1β protein levels^42,47–50^. Furthermore, engulfment of *S. aureus* by polymorphonuclear cells (PMNs) is known to alter macrophage production of IL-6, which subsequently lowers secretion of IL-1β^49^. Our cytokine results indicated that the host response to LA-*S. aureus* infections might be suboptimal, as LA-*S. aureus-*infected mice had larger lesions, higher bacterial burden and decreased IL-1β and MIP2 and increased IL-6 levels compared with SF8300-infected mice. Neutrophil recruitment to the site of *S. aureus* infection in the skin is an event required for bacterial clearance^47,48,51^, and, based on our cytokine data, may be attenuated among LA-*S. aureus* infected mice. A possible explanation for these findings is that LA-*S. aureus* have a distinct VF repertoire that might have suppressed the optimal IL-1β neutrophilic response required for bacterial clearance of *S. aureus* from the site of the SSTI. *S. aureus* alpha-toxin, for example, has been shown to suppress local IL-1β production in a mouse model of SSTI^42^. Future studies should include abscess and skin pathology, which could help in characterizing the recruitment of immune cell populations to the site of infection.

Although all the isolates displayed phenotypic hemolysis, our WGSA revealed that only LA-*S. aureus* CC398 and CC9 carried an intact beta-hemolysin gene. An insertion of the Sa3 prophage into the beta-hemolysin gene^52^ results in a truncated beta-hemolysin gene in both of the CC8 genomes (SF8300 and NCHW8). A previous study has reported that mutant strains lacking the Sa3 prophage, thereby carrying an intact beta-hemolysin gene, display a greater degree of hemolysis compared to its wild type^53^. An intact *hlb* gene has been previously shown to increase *S. aureus* fitness for colonization and persistence in mice^53^. However, a critical role for *hlb* in the pathogenesis of SSTI is not entirely clear^41^. The gain of an intact beta-hemolysin gene is dependent on the loss of human immune evasion cluster (IEC) genes – the Sa3 prophage can carry several human IEC genes including *scn* and *sak*^52^, which were absent from the genomes of the LA-*S. aureus* CC398 and CC9 isolates (IHW398-1, IHW398-2, and NCHW9). The staphylococcal complement inhibitor protein has been reported to be highly immunogenic^54^ and functional only against components of the human immune system^52^. A loss of the Sa3 prophage and its associated proteins may provide improved virulence for LA-*S. aureus* CC398 in a mouse model of SSTI. Similar to IEC encoded proteins, leukocidins largely lack activity in mice^45^ but are highly immunogenic^55,56^. MGE encoded leukocidin genes^57^ *lukE* and *lukD*, encoding for LukED, and *lukS-PV* and *lukF-PV*, encoding for PVL, were absent from the LA-*S. aureus* CC398 and CC9 genomes. Thus, the loss of MGE encoded leukocidins may also provide improved virulence in a mouse model of SSTI. The *nor* gene encoding for a nitric oxide reductase enzyme, commonly associated with pathogenic bacteria, and hypothesized to provide improved virulence and fitness for *S. aureus* infections *in vivo*^40^, was also only found among the LA-*S. aureus* CC398 isolates that produced the largest lesions (IHW398-1 and IHW398-2). Taken together, multiple genetic differences between LA- and CA- *S. aureus*, including the gain or loss of a combination genes encoding for important hemolytic toxins, leukocidins, and immune evasion proteins, could play a role in the observed pathogenicity of LA- and CA- *S. aureus* SSTI in mice.

Our study had several strengths. First, this was to our knowledge, the first experimental design involving LA-*S. aureus* strains isolated from nasal carriage and SSTI events of IHO workers in the United States. Second, this study was the first, to our knowledge, to characterize the relative pathogenicity of a LA-*S. aureus* CC9 strain, which appears to be an emerging clone among IHO workers in North Carolina and Asia^7,10,58^. Finally, we compared all LA-*S. aureus* strains to a well-characterized USA300 CC8 strain that has consistently demonstrated substantial pathogenicity in C57BL/6 mouse models of SSTI and capacity to cause human infections in community settings^29,30^.

Our study also has several limitations. First, certain *S. aureus* virulence factors (VFs) expected to increase pathogenicity may have low, or no activity in a mouse model of SSTI, such as PVL, HlgAB, and HlgBC^45^. Furthermore, LA-*S. aureus* largely do not carry human immune evasion cluster (IEC) genes, *scn* and *sak*^52^, suggesting a host adaptation to animals^13^. Thus, these results should be interpreted within the limitations of using a mouse model to compare the virulence of the included *S. aureus* strains. Finally, our study characterizes the pathogenicity of a single LA-*S. aureus* nasal carriage isolate, whereas isolates within the LA-*S. aureus* CC398 and CC9 lineages display considerable diversity. Thanh-Thao *et al*. reports a conservation of core-genome elements, such as *spa type*, between nasal carriage isolates within individual, but more heterogeneity between isolates for mobile genetic element (MGE) encoded genes, such as *scn* and AMR genes^59^. Differences in pathogenicity between closely related isolates of *S. aureus* is well established, and future studies should compare multiple isolates of each lineage to improve confidence in lineage related pathogenicity conclusions.

## Conclusions

The increased pathogenicity and bacterial burden associated with LA-*S. aureus* skin infection compared to a highly virulent CA-MRSA skin infection raises both occupational and public health concerns of critical importance. Multiple epidemiologic studies have concluded that exposure to LA-*S. aureus* is associated with SSTI among IHO workers and their household contacts^4,7,9,10,27^, and community residents with no known exposure to livestock^10,11^. Thus, our pathogenicity findings suggest that infections with LA-*S. aureus*, which are not limited to the occupational setting, could represent a broader public health concern. Particularly concerning is that these LA-*S. aureus* strains are largely multidrug-resistant^4,8,10^, which may have clinical implications regarding treatment of LA-*S. aureus* infections. The results of this study provide new insights into the pathogenicity of emerging LA-*S. aureus* strains that are commonly contracted by IHO workers and emerging among populations with no known livestock exposure. Mice infected with LA-*S. aureus* displayed increased or equivalent pathogenicity in a mouse model of SSTI compared to USA300 clone, SF8300, marked by larger lesion sizes and higher bacterial burden. IL-1β signaling was significantly diminished among LA-*S. aureus* infected mice compared to CA-MRSA infected mice, suggesting suboptimal immune cell recruitment for bacterial clearance during LA-*S. aureus* infections. WGSA suggests that a subset of VF genes involved in the pathogenesis of *S. aureus* SSTIs were present among CA-MRSA and LA-*S. aureus* isolates, while LA-*S. aureus* isolates carried a greater frequency and diversity of AMR genes encoding for resistance to antibiotics that are critically important for human health^60^. Therefore, future research efforts should be considered to prevent exposure to LA-*S. aureus* strains and study, monitor, and determine virulence mechanisms, host immune responses and response to treatment in humans that suffer SSTI caused by LA-*S. aureus* strains.

## Supplementary information

Supplementary Information

## Data Availability

The datasets generated during and/or analyzed during the current study are available from the corresponding author upon request.
