# Author Correction: Comparison of livestock-associated and community-associated *Staphylococcus aureus* pathogenicity in a mouse model of skin and soft tissue infection

**DOI:** 10.1038/s41598-019-46940-z

**Published:** 2019-09-02

**Authors:** Pranay R. Randad, Carly A. Dillen, Roger V. Ortines, David Mohr, Maliha Aziz, Lance B. Price, Hülya Kaya, Jesper Larsen, Karen C. Carroll, Tara C. Smith, Lloyd S. Miller, Christopher D. Heaney

**Affiliations:** 1 Department of Environmental Health and Engineering, Johns Hopkins Bloomberg School of Public Health, Baltimore, Maryland USA; 2 Department of Dermatology, Johns Hopkins School of Medicine, Baltimore, Maryland USA; 3 Genetic Resources Core Facility, McKusick-Nathans Institute of Genetic Medicine, Johns Hopkins University School of Medicine, Baltimore, Maryland USA; 4 Department of Environmental and Occupational Health, George Washington University, Washington, D.C. USA; 5 Antibiotic Resistance Action Center, George Washington University, Washington, D.C. USA; 6 Department of Bacteria, Parasites and Fungi, Statens Serum Institut, Copenhagen, Denmark; 7 Division of Medical Microbiology, Johns Hopkins University School of Medicine, Baltimore, Maryland USA; 8 Department of Epidemiology and Biostatistics, Kent State University, Kent, Ohio, USA; 9 Department of Epidemiology, Johns Hopkins Bloomberg School of Public Health, Baltimore, Maryland USA; 10 Department of International Health, Johns Hopkins Bloomberg School of Public Health, Baltimore, Maryland USA

Correction to: *Scientific Reports* 10.1038/s41598-019-42919-y, published online 01 May 2019

In the original version of this manuscript, the incorrect isolate ID was provided for NCHW8.

In addition, the Article contained an error in Affiliation 6, which was incorrectly given as ‘Department of Bacteria, Parasites and fungi, Staten Serum institut, copenhagen, Denmark’. The correct affiliation is listed below:

Department of Bacteria, Parasites and Fungi, Statens Serum Institut, Copenhagen, Denmark

These errors have now been corrected in the HTML and PDF versions of the article.

