# Supplementary material for: Comparison of livestock-associated and community-associated Staphylococcus aureus pathogenicity in a mouse model of skin and soft tissue infection
Source: Sci Rep. 2019 May 1;9:6774. doi: 10.1038/s41598-019-42919-y (PMC6494861; doi:10.1038/s41598-019-42919-y)
Supplement: Supplementary file 1 — Supplementary Information [file 41598_2019_42919_MOESM1_ESM.pdf]

Supplemental Material

Comparison of livestock-associated and community-associated *Staphylococcus aureus* pathogenicity in a mouse model of skin and soft tissue infection

Pranay R. Randad*[1], Carly A. Dillen[2], Roger O. Ortines[2], David Mohr[3], Maliha Aziz[4,5], Lance B. Price[4,5], Hülya Kaya[6], Jesper Larsen[6], Karen C. Carroll[7], Tara C. Smith[8], Lloyd S. Miller[2], Christopher D. Heaney*[1,9,10]

Table S1. Antibiotics used, and MIC cut-off values, for susceptibility testing of *S. aureus* isolates.

<u>Table S1</u>. Antibiotics used, and MIC cut-off values, for susceptibility testing of *S. aureus* isolates.

Antimicrobial Agent	Susceptible (µg/mL)	Intermediate (ug/mL)	Resistant (µg/mL)
**Penicillin (PEN)**	</=0.2	N/A	>/=.25
**Erythromycin (ERY)**	</=0.5	1.0-4.0	>/= 8
**Clindamycin (CLIN)**	0.5	1.0-2.0	>/= 4.0
**Moxifloxacin (MOXI)**	</=0.5	1	>/= 2.0
**Tetracycline (TET)**	</=4	8	>/=16.0
**Trimethoprim/sulfamethoxazole (TRI/SUL)**	</=2/38	N/A	>/=4/76
**Gentamycin (GEN)**	</=4	8	>/=16
**Cefoxitin (CEFO)**	</=4	N/A	>/=4
**Oxacillin (OXA)**	</= 0.25	N/A	>/=0.5
**Quinupristin/dalfopristin (QUI/DALF)**	</=1	2	>/=4
**Minocycline (MINO)**	</=4	8	>/=16.0
**Nitrofurantoin (NIT)**	</=32	64	>/=16
**Rifampin (RIF)**	</=1	2	>/=4
**Linezolid (LIN)**	</=4	N/A	>/=8
**Daptomycin (DAP)**	</=1	N/A	N/A
**Vancomycin (VAN)**	</=2	4.0-8.0	>/= 16
